# Rapid isothermal duplex real-time recombinase polymerase amplification (RPA) assay for the diagnosis of equine piroplasmosis

**DOI:** 10.1038/s41598-020-60997-1

**Published:** 2020-03-05

**Authors:** Rong Lei, Xinyi Wang, Di Zhang, Yize Liu, Qijun Chen, Ning Jiang

**Affiliations:** 1 Key Laboratory of Livestock Infectious Diseases in Northeast China, Ministry of Education, Key Laboratory of Zoonosis, Shenyang Agricultural University, Shenyang, 110866 China; 2 Chinese Academy of Inspection and Quarantine, Beijing, 100176 China; 3 The Research Unit for Pathogenic Mechanisms of Zoonotic Parasites, Chinese Academy of Medical Sciences, 120 Dongling Road, Shenyang, 110866 China; 4 College of Land and Environment, Shenyang Agricultural University, Shenyang, 110866 China

**Keywords:** Microbiology, Microbiology, Molecular biology, Molecular biology

## Abstract

Equine piroplasmosis (EP) is a severe disease of horses caused by the tick-borne protozoa *Theileria equi* (*T. equi*) and *Babesia caballi* (*B. caballi*). Infectious carriers are not always symptomatic, meaning there is a risk to non-enzootic areas. Regulatory tests for EP include sero-epidemiological methods for equine babesiosis, but these lack specificity due to cross-reactivity with other *Babesia* species. In this study, we present a real-time quantitative recombinase polymerase amplification (qRPA) method for fast simultaneous detection of both *T. equi* and *B. caballi*. In this method, primers and probes targeting the *18S rRNA* gene of both *T. equi* and *B. caballi*, the *ema-1* gene of *T. equi* and the *bc48* gene of *B. caballi* were designed and evaluated. The sensitivity of qRPA was evaluated using the pUC57 plasmid DNA containing the target gene. For the pUC57-bc48 gene DNA, the R^2^ value was 0.983 for the concentration range 0.2 ng (4.1 × 10^7^ DNA copies) to 2.0 fg (4.1 × 10^1^ DNA copies). For the pUC57-*ema* gene DNA, the R^2^ value was 0.993 for the concentration range 0.2 ng (5.26 × 10^7^ DNA copies) to 2.0 fg (5.26 × 10^2^ DNA copies). For the pUC57-Bc18S gene DNA the R^2^ value was 0.976 for the concentration range 2.0 ng (4.21 × 10^8^ DNA copies) to 2.0 fg (4.21 × 10^2^ DNA copies). For the pUC57-Te18S gene DNA, the R^2^ value was 0.952 (Fig. S3b) for the concentration range 2.0 ng (4.16 × 10^8^ DNA copies) to 2.0 fg (4.16 × 10^2^ DNA copies). Furthermore, a duplex qRPA analysis was developed and optimized and the results showed that primers and probes targeting for the *bc48* gene of *B. caballi* and the *18S rRNA* gene of *T. equi* is the best combination for a duplex qRPA analysis in one reaction. The developed duplex qRPA assay has good specificity, and had negative amplification for several similar parasite. For DNA extracted from real horse blood specimens, this qRPA method has comparable sensitivity to traditional qPCR, but a simpler and more rapid operating process to obtain positive amplification. The qRPA, including the duplex strategy described here, could allow fast identification of the EP-causing *T. equi* and *B. caballi*, showing great potential for on-site EP screening of horses.

## Introduction

**Figure 1:**
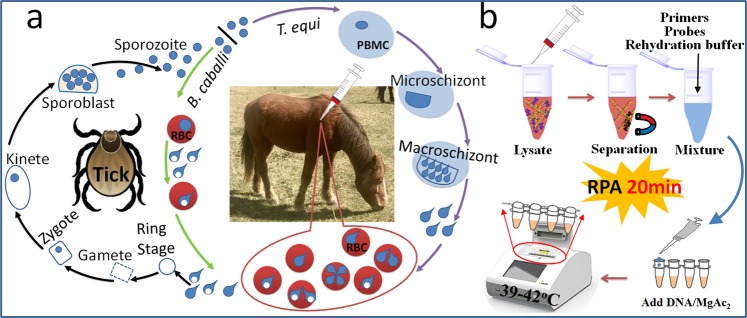
Schematic description of a duplex real-time RPA strategy for the diagnosis of *B. caballi* and *T. equi* in horse blood specimens. (**a**) Life cycle of *B. caballi* and *T. equi* within the tick vector and within the horse host. PBMCs: peripheral blood mononuclear cells; RBCs: red blood cells. (**b**) Method of rapid genomic DNA identification and RPA reaction.

Equine piroplasmosis (EP) is a haemoprotozoan infection of horses and other members of the Equidae family. It is caused via tick transmission of two intra-erythrocyte protozoa, *Theileria equi* (also called *Babesia equi*) and *Babesia caballi* (*B. caballi*)^1–3^. There are differences between *B. caballi* and *T. equi* in both the tick vector and the horse host, which is reflected in disease severity and drug susceptibility (Fig. 1a). However, making a pathogenic diagnosis is still difficult as the parasites have the same vector and a similar clinical presentation, and in addition cross-infection is possible.

Infected animals present with severe acute disease characterized by high fever, lethargy, anorexia, peripheral oedema, splenomegaly, haemolysis, tachycardia, pigmenturia and occasionally death^1^. Animals who recover from primary infection remain recessive carriers with fluctuating levels of parasitaemia^4,5^. This disease is widely distributed in Asia, Europe, Africa and South America, causing economic loss and impacting the international movement of horses^6,7^. Importation of carrier animals with no obvious signs of disease is a major risk factor for the introduction of EP into non-enzootic areas^1^. Therefore, developing sensitive and specific diagnostic methods is essential for identifying asymptomatic equines carrying these parasites.

Current diagnostic methods include aetiological diagnostics^8,9^, immunological diagnostics^10–14^ and molecular diagnostics^9,15–33^. Of these, molecular diagnostics is widely recognized due to its accuracy and sensitivity^9,34^. Cortes *et al*. developed a multinested PCR assay for simultaneous detection of the equine piroplasmids *T. equi* and *B. caballi* by amplification of five genetic markers (*18S rRNA*, *β-tubulin*, *cytB*, *ema-1* and *rap-1*)^23^. To identify the species of piroplasmid in an infected horse, nested PCR assay is commonly used to amplify long fragments of *18S rRNA*, followed by sequence analysis^22^. However, PCR-based diagnosis requires a thermocycler, skilled personnel and a long detection time, which made it inappropriate for field diagnostic applications. As an alternative, recombinase polymerase amplification (RPA) has emerged as a novel isothermal technique for molecular diagnosis of various infectious diseases^35^, including the protozoan parasites^36^ *Theileria annulata*^37^ and *Babesia gibsoni*^38^. Compared to PCR-based assay and loop-mediated isothermal amplification (LAMP), RPA is more rapid (<20 min), simpler to perform as it requires a lower temperature (37–42 °C) and has an acceptable sensitivity^39,40^. RPA-lateral flow assay has been used for rapid detection of *Trichinella*^41^, *Perkinsus beihaiensis*^42^, *Plasmodium knowlesi*^43^, *Babesia gibsoni*^38^, *Protozoan parasites*^36^, *Theileria annulata*^37^, *Fasciola hepatica*^44^, *Schistosoma japonicum*^45^, *Schistosoma haematobium*^46^, *Leishmania donovani*^47^, *Intestinal Protozoa*^48^, *Giardia*^49^, *Plasmodium falciparum*^50^. To avoid the “ghost band” and the false-positive results, the primers, probes and the detection procedure have to be carefully designed^39^.

To speed up the clearance of an imported horse at a port, or detect a piroplasmosis infection in the field or in low-resource underserved rural communities, we also adapted RPA to develop a duplex detection of both *T. equi* and *B. caballi* as an alternative to duplex qPCR^26,30^. Various genomic sites have been used in species identification, phylogenetic and genotype studies of both *T. equi* and *B. caballi* with PCR-based molecular techniques, including the small subunit ribosomal RNA gene (*18S rRNA*)^11,22,51–54^; and genomic sites targeted by qPCR assay have included the *ema-1* gene of *T. equi* and the 48 kDa merozoite rhoptry protein (*bc48*) gene of *B. caballi*^30^. These sites have been used to create a duplex real-time PCR for simultaneous detection of both parasites using the *ema-1* gene of *T. equi* and the *bc48* gene of *B. caballi*^30^; or the *18S rRNA* gene of *B. caballi* and the *ema-1* gene of *T. equi*^26^. In this study, we designed primers and fluorescent probes to target the *18S rRNA* gene of both *T. equi* and *B. caballi*, the *ema-1* gene of *T. equi* and the *bc48* gene of *B. caballi*. A secondary aim was to investigate the combination of target genes to construct the duplex assay.

## Results and Discussion

### Duplex real-time RPA strategy for the diagnosis of equine piroplasmosis

A molecular diagnostic method based on duplex real-time RPA for the diagnosis of both *B. caballi* and *T. equi* from infected horse blood was developed (Fig. 1b). To achieve rapid diagnosis, specimen DNA extraction and separation were performed using magnetic beads, as an alternative to traditional high-speed centrifugation. To simplify the steps, all reagents (including extracted DNA, primers, probes, freeze-dried enzymes and rehydration buffer) were one-time mixed and the amplification reaction was initiated by MgAc_2_ reagent at 37–42 °C. During RPA amplification, the fluorescent signal was created using an oligonucleotide probe flanked by a dT-fluorophore and a corresponding dT-quencher group, and was observed with a portable fluorescence detection device. Therefore, this strategy allows rapid diagnosis of piroplasmosis on-site.

### Primer design

**Figure 2:**
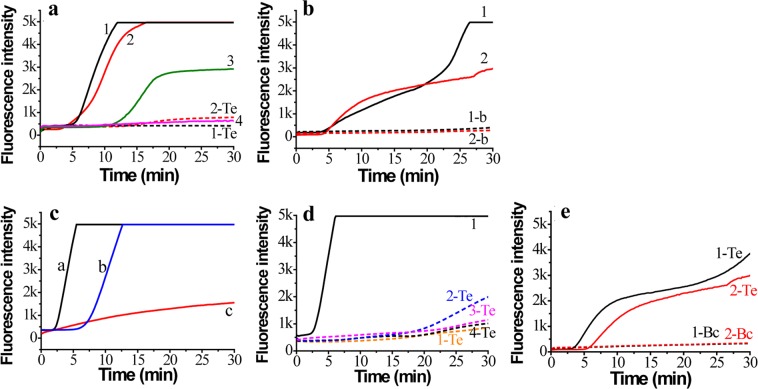
Real-time fluorescence curves of RPA assay using different primers and probes. (**a**) B. caballi-bc48-F1/R2 (line 1) and Primer B. caballi-bc48-F2/R1 (line 2), targeting the *B. caballi bc48* gene for pUC57-bc48 plasmid DNA; (**b**) Primer T. equi-ema-F1/R1 (line 1) and T. equi-ema-F2/R2 (line 2), targeting *T. equi* ema-1 gene for pUC57-ema plasmid DNA. (**c**) Primer B. caballi-Bc18S-F1/R1 assay for pUC57-Bc18S plasmid DNA (line a), pUC57-Te18S plasmid DNA (line b) and T. equi-Bc18S-F1/R1 assay for pUC57-Bc18S plasmid DNA (line c); (**d**) Primer B. caballi-Bc18S-F2/R2 for pUC57-Bc18S plasmid DNA (line 1), pUC57-Te18S plasmid DNA (line 1-Te), B. caballi-Bc18S-F2/R3 (line 2-Te), B. caballi-Bc18S-F3/R2 (line 3-Te) and B. caballi-Bc18S-F3/R3 (line 4-Te) for pUC57-Te18S plasmid DNA; (**e**) Primer T. equi-18S-F2/R2 for pUC57-Te18S plasmid DNA (line 1-Te), pUC57-Bc18S plasmid DNA (line 1-Bc), T. equi-18S-F3/R2 for pUC57-Te18S plasmid DNA (line 2-Te), and pUC57-Bc18S plasmid DNA (line 2-Bc).

Four primers targeting the *bc48* gene of *B. caballi* and the *ema-1* gene of *T. equi* were tested for sensitivity and specificity. The B. caballi-bc48-F1/R2 primer could amplify pUC57-bc48 (Fig. 2a, line 1), but not *T. equi* genomic DNA (Fig. 2a, line 1-Te). The B. caballi-bc48-F2/R1 primer was able to amplify pUC57-bc48 (Fig. a, line 2) and *T. equi* genomic DNA to some extent (Fig. 2a, line 2-Te). The B. caballi-bc48-F1/R1 primer produced an amplification curve with only water as the template (Fig. 2a, line 3). The primer set B. caballi-bc48-F2/R2 has a low amplification efficiency for *T. equi* genomic DNA (Fig. 2a, line 4). Therefore, B. caballi-bc48-F1/R2 was chosen to test the sensitivity for pUC57-bc48 and *B. caballi* genomic DNA.

Additionally, two primer sets (T. equi-ema-F1/R1 and T. equi-ema-F2/R2) could amplify pUC57-ema DNA (Fig. 2b, lines 1&2), but not *B. caballi* genomic DNA (Fig. 2b, lines 1-b and 2-b). Therefore, both primers could be used for pUC57-ema DNA and *T. equi* genomic DNA.

In this study, we also designed the primers and probes for the *18S rRNA* gene of *B. caballi* and *T. equi*. Because of the high similarity of this gene between the two vectors, neither B. caballi-18S-F1/R1 nor T. equi-18S-F1/R1 were able to specifically amplify the plasmid DNA (Fig. 2c). We found that neither B. caballi-18S-F2/R2 (Fig. 2d, line 1-Te), nor B. caballi-18S-F3/F2 (Fig. 2d, line 3-Te) nor B. caballi-18S-F3/R3 (Fig. 2d, line 4-Te) were able to amplify the *T. equi 18S rRNA* gene; although B. caballi-18S-F2/R3 could amplify this gene to some extent (Fig. 2d, line 2-Te). Neither T. equi-18S-F2/R2 nor T. equi-18S-F3/R2 were able to amplify the *B. caballi 18S rRNA* gene, although T. equi-18S-F2/R2 showed good amplification for pUC57-T.equi-18S plasmid DNA (Fig. 2e). Therefore, B. caballi-18S-F2/R2, B. caballi-18S-F3/F2, B. caballi-18S-F3/R3, T. equi-18S-F2/R2 and T. equi-18S-F3/R2 primers could be used for a pUC57-Bc18S or pUC57-Te18S plasmid DNA assay.

### Duplex RPA assay

**Figure 3:**
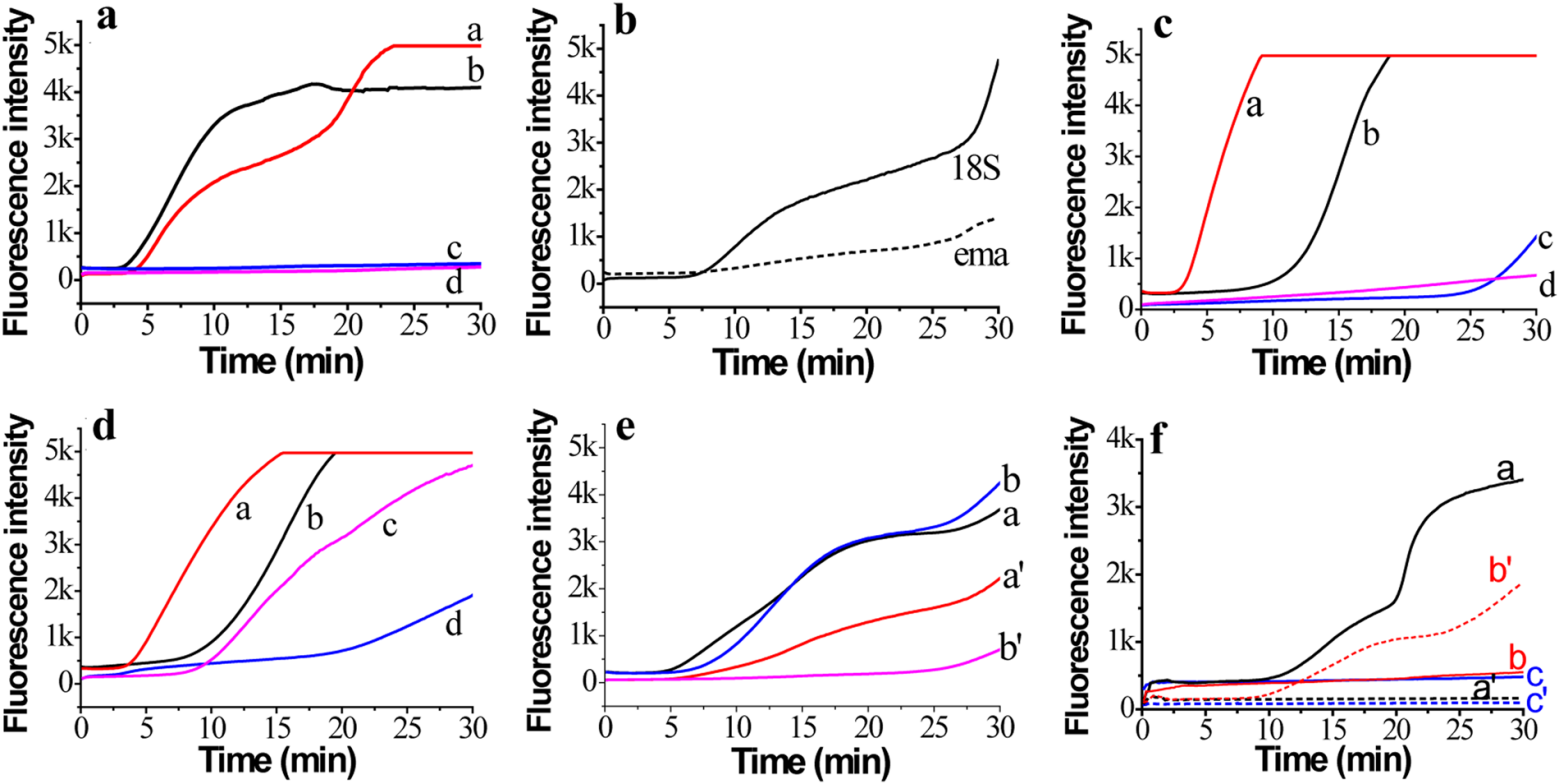
Duplex real-time RPA assay. (**a**) Assay using a mixture of primer B. caballi-bc48-F1/R2 and T. equi-ema-F1/R1 for pUC57-bc48 plasmid DNA (lines b, c) and pUC57-ema plasmid DNA (lines a, d). (**b**) Assay for *T. equi* genomic DNA using primer T. equi-18S-F3/R2 (solid line) and T. equi-ema-F1/R1 (short dashed line). (**c**) T. equi-18S-F3/R2/B. caballi-18S-P for pUC57-Te18S plasmid DNA (line a) and pUC57-Bc18S plasmid DNA (line b); B. caballi-18S-F3/R3/T.equi-18S-P for pUC57-Bc18S plasmid DNA (line c) and pUC57-Te18S plasmid DNA (line d). (**d**) T. equi-18S-F2/R2/B.caballi-18S-P for pUC57-Te18S plasmid DNA (line a) and pUC57-Bc18S plasmid DNA (line b); B. caballi-18S-F2/R2/T. equi-18S-P for pUC57-Te18S plasmid DNA (line c) and pUC57-Bc18S plasmid DNA (line d). (**e**) Mixture of T. equi-18S-F3/R2 and B. caballi-18S-F3/R2 for pUC57-Te18S plasmid DNA (lines a, a’) or pUC57-Bc18S plasmid DNA (lines b, b’). (**f**) Mixture of T. equi-18S-F2/R2 and B. caballi-Bc48-F1/R2 for *B. caballi* genomic DNA (lines, a, a’), *T. equi* genomic DNA (lines, b, b’) and water (lines, c, c’).

To develop the duplex RPA assay for *B. caballi* and *T. equi*, we first investigated the primers and probes targeting the *bc48* gene of *B. caballi* and the *ema-1* gene of *T. equi* using pUC57-bc48 and pUC57-ema plasmid DNA. A carboxy fluorescein (FAM)-signal was only detected from pUC57-bc48 plasmid DNA (Fig. 3a, lines b& d); and a ROX-signal was only detected from pUC57-ema plasmid DNA (Fig. 3a, lines a&c). This indicated good specificity of these primers and probes. Compared with the primers and probes targeting the *ema-1* gene, those targeting the *18S rRNA* gene of *T. equi* had greater amplification (Fig. 3b), possibly due to *ema-1* gene sequence heterogeneity in a subset of the original isolates^27^. Therefore, we chose T. equi-18S-F2/R2 with a T. equi-18S-probe to amplify *T. equi* genomic DNA.

When the primers and probes targeting the *18S rRNA* genes of *B. caballi* and *T. equi* were mixed in one reaction tube, the amplification signals showed cross interference (Fig. 3c–e), which meant that developing a duplex assay based on these primers and probes was inappropriate. Therefore, primers B. caballi-bc48-F1/R2 and T. equi-18S-F2/R2 were chosen to establish a duplex RPA strategy for *B. caballi* and *T. equi*. These primers and probes were added into one reaction tube to detect water (Fig. 3f, blue lines), *B. caballi* genomic DNA (Fig. 3f, black lines) and *T. equi* genomic DNA (Fig. 3f, red lines). Results showed specific amplification of *B. caballi* or *T. equi* genomic DNA and no cross interference. Therefore, a valid duplex RPA strategy was developed by employing a B. caballi-bc48-F1/R2/B. caballi-bc48-probe and a T. equi-18S-F2/R2/T. equi-18S-probe for the diagnosis of equine piroplasmosis.

### Sensitivity and specificity of real-time RPA assay

**Figure 4:**
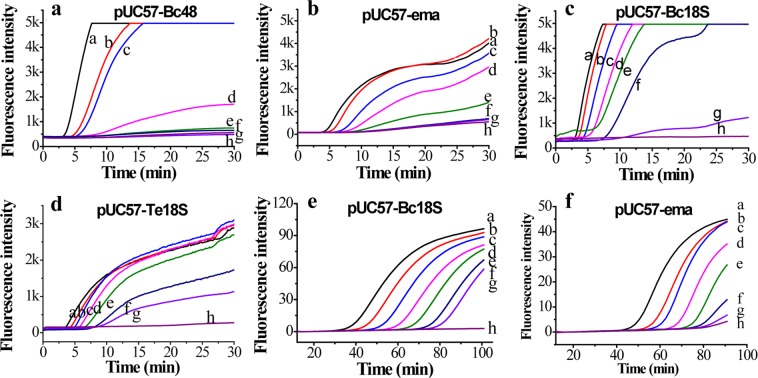
Sensitivity of real-time RPA assay. (**a**) pUC57-Bc48 plasmid DNA with primer B. caballi-bc48-F1/R2. (**b**) pUC57-ema plasmid DNA with primer T. equi-ema-F1/R1; lines a-h, from 0.2 ng to 0.02 fg. (**c**) pUC57-Bc18S plasmid DNA with primer B. caballi-Bc18S-F3/R2. (**d**) pUC57-Te18S plasmid DNA with primer T. equi-18S-F3/R2/; lines a-g, from 2 ng to 2 fg. Line h, water. (**e**) Real-time PCR assay for serially-diluted pUC57-Bc18S plasmid DNA; (**f**) Real-time PCR assay for serially-diluted pUC57-ema plasmid DNA; lines a–g, from 2 ng to 2 fg. Line h, water.

To evaluate the sensitivity of the primers and probes for the *bc48* gene of *B. caballi* and the *ema-1* gene of *T. equi*, we chose B. caballi-bc48-F2/R2 and T. equi-ema-F1/R1 to evaluate the sensitivity and linearity of the real-time RPA assay. The amplification curves are shown in Fig. 4a,b. For the pUC57-bc48 gene DNA, the R^2^ value was 0.983 (Fig. S2a) for the concentration range 0.2 ng (4.1 × 10^7^ DNA copies) to 2.0 fg (4.1 × 10^1^ DNA copies). For the pUC57-*ema* gene DNA, the R^2^ value was 0.993 (Fig. S2b) for the concentration range 0.2 ng (5.26 × 10^7^ DNA copies) to 2.0 fg (5.26 × 10^2^ DNA copies).

The sensitivity of the primers and probes for the *18S rRNA* gene of *B. caballi* and *T. equi* assay were evaluated using B. caballi-18S-F3/R2 and T. equi-18S-F3/R2 respectively (Fig. 4c,d). The linearity results indicated that for the pUC57-Bc18S gene DNA the R^2^ value was 0.976 (Fig. S3a) for the concentration range 2.0 ng (4.21 × 10^8^ DNA copies) to 2.0 fg (4.21 × 10^2^ DNA copies). For the pUC57-Te18S gene DNA, the R^2^ value was 0.952 (Fig. S3b) for the concentration range 2.0 ng (4.16 × 10^8^ DNA copies) to 2.0 fg (4.16 × 10^2^ DNA copies).

Real-time PCR is the widely-recognized gold standard of genomic DNA identification and quantification due to its good specificity and high sensitivity. In this study, we employed the reported PCR primers and probes to assay pUC57-Bc18S and pUC57-ema DNA^26,28^ and the results showed that 2.0 fg pUC57-Bc18S DNA (Figs. 4e) and 2.0 fg of pUC57-ema DNA (Fig. 4f) could be detected. The onset of real-time PCR was linearly fitted against the logarithm of initial pUC57-Bc18S concentration from 2.0 fg (4.21 × 10^2^ DNA copies) to 2.0 ng (4.21 × 10^8^ DNA copies), with an R^2^ value of 0.998 (Fig. S4a); and for pUC57-ema concentration from 2.0 fg (5.26 × 10^2^ DNA copies) to 2.0 ng (5.26 × 10^8^ DNA copies), with a R^2^ value of 0.997 (Fig. S4b). This shows that the sensitivity of the developed real-time RPA is comparable to real-time PCR assay.

**Figure 5:**
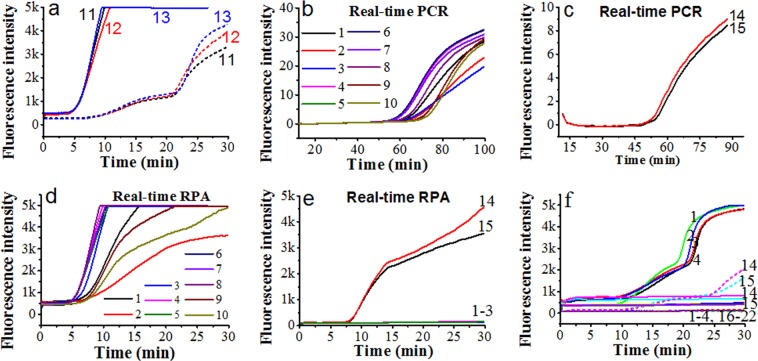
Fluorescence curves of horse blood assay using real-time PCR and real-time RPA methods. (**a**) Real-time RPA assay for *B. caballi* genomic DNA using primer B. caballi-bc48-F1/R2/bc48-P (solid lines, three samples) and B. caballi-18S-F3/R2/bc-18S-P (short dashed lines, three samples). Line 11, a; Line 12, b; Line 13, c. (**b**) Real-time PCR assay for real samples containing *B. caballi* genomic DNA using Bc_18SF402/ c_18SR4596/Bc_18SP. Lines 1–10, horse blood samples, numbered HQ1–10. (**c**) Real-time PCR assay for real samples containing *T. equi* genomic DNA using Te_EMA1-F/ Te_EMA1-R/Te_EMA1-P. Lines 14 and 15, horse blood samples, numbered DQ1 and DQ2. (**d**) Real-time RPA assay for real samples containing *B. caballi* genomic DNA using B. caballi-bc48-F1/R2/bc48-P. Lines 1–10, HQ 1–10. (**e**) Real-time RPA assay for real samples containing *T. equi* genomic DNA using T. equi-18S-F2/R2/T. equi-18S-P. Line 14, DQ1; line 15, DQ2; lines 1–3, HQ 1–3. (**f**) Real-time fluorescence curves of a duplex assay for *B. caballi* genomic DNA (line 1, HQ1; 2, HQ2; 3, HQ-3; 4, HQ-4), *T. equi* genomic DNA (line 14, DQ1; 15, DQ2), *trypanosoma evansi* (line 16), *trypanosome brucei* (line 17), *toxoplasma gondii* (line 18), *plasmodium falciparum* (line 19), *theileria hirci* (line 20), *theileria sinensis* (line 21), *trichinella spiralis inorganic* (line 22). Solid lines indicated the FAM signal generated by the *bc48* gene of *B. caballi*, and short dashed lines indicated the ROX signal generated by the *18S* gene of *T. equi*.

The specificity of the developed qRPA was evaluated using several other parasite (Sample No. 16–22 in Table 1), and the results (Fig. 5f) showed that none of these parasites generated FAM or ROX fluorescence signals in the duplex RPA reaction containing the primers and probes targeting for *bc48* gene of *B. caballi* and the *18S gene* of *T. equi*. But the *B. caballi* genomic DNA from sample HQ1–4 could generate remarkable fluorescence signals of FAM Dye and *T. equi* genomic DNA from sample DQ1 and DQ2 produced obvious fluorescence signals of ROX Dye (Fig. 5f), indicating that the developed primers and probes have good specificity.

**Table 1:** Information of parasites studied in this study.

No.	Strain code	Species	Con. (ng/μL)	Source
1	HQ1	*B. caballi*	2.88	From the Hongshan Military Horse Farm of the Beijing Military Region in the Keshiketeng Banner, Chifeng, Inner Mongolia
2	HQ2	*B. caballi*	2.92	
3	HQ3	*B. caballi*	2.925	
4	HQ4	*B. caballi*	2.215	
5	HQ5	*B. caballi*	2.1	
6	HQ6	*B. caballi*	2.93	
7	HQ7	*B. caballi*	3.01	
8	HQ8	*B. caballi*	2.83	
9	HQ9	*B. caballi*	2.91	
10	HQ10	*B. caballi*	2.88	
11	a	*B. caballi*	4.13	
12	b	*B. caballi*	4.20	
13	c	*B. caballi*	4.16	
14	DQ1	*T.equi*	3.61	Heilongjiang Bayi Agricultural University, Daqing, Heilongjiang
15	DQ2	*T.equi*	3.06	
16	STIB 805	*trypanosoma evansi*	14.0	Shenyang Agricultural University, Shenyang, Liaoning
17	TREU927	*trypanosome brucei*	28.0	
18	RH	*toxoplasma gondii*	5.0	
19	3D7	*plasmodium falciparum*	5.0	
20	Dschunkowsky	*theileria hirci*	1.7	
21	TS1	*theileria sinensis*	5.17	
22	TSI 1	*Trichinella spiralis*	1.5

### Applicability of RPA assay for horse blood samples

To investigate the efficacy of the developed primers and method in a real sample, genomic DNA extracted from horse blood containing *B. caballi* or *T. equi* was used as a template for an RPA assay. Horse blood samples collected from the Hongshan Military Horse Farm of the Beijing Military Region during a period of high incidence of equine piroplasmosis in April, 2018 were detected. Before detection, we evaluated the amplification efficiency of primer sets (the *18S rRNA*, *bc48* and *ema-1* genes). For *B. caballi*, the *bc48* gene primers had better amplification efficiency than the *18S* gene primers (Fig. 5a). For *T. equi*, the *18S* gene primers had better amplification efficiency than the *ema-1* gene primers (Fig. 3b). Therefore, B. caballi-bc48-F1/R2 and T. equi-18S-F2/R2 were applied to assay the real horse blood sample.

The horse blood samples containing *B. caballi* or *T. equi* were assayed using real-time RPA (Fig. 5d,e), and the results compared to real-time PCR (Fig. 5b,c). Results of all the samples were consistent according to the onset time of the two methods. Furthermore, the chosen specimens containing *B. caballi* were assayed using the developed duplex real-time RPA method. The results showed that only FAM signals were generated from *B. caballi* samples (Fig. 5f), confirming the validity of the developed method for diagnosing EP. The new method has the advantages of being more rapid, whilst maintaining high sensitivity and specificity.

### Repeatability of RPA assay

**Figure 6:**
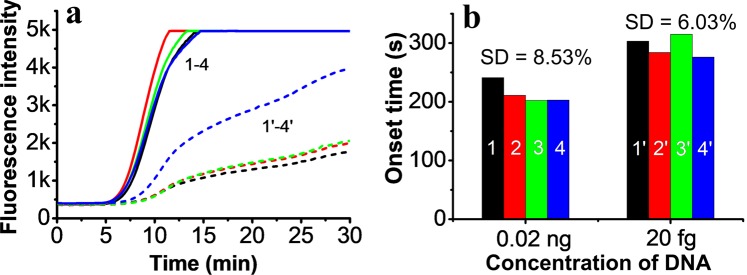
Repeatability of the RPA assay. (**a**) The real-time fluorescence curves of the RPA assay for 0.02 ng (solid line, four samples) and 20 fg (short dashed lines, four samples) of pUC57-bc48 plasmid DNA. (**b**) The onset time of real-time amplification of four assays. The colour of the columns corresponds to the colour of the lines in part (**a**).

During RPA amplification, fluorescence intensity can easily be affected by the concentration of the probe and DNA template, even by the pipette due to the small volume of DNA template^40^. The repeatability of the RPA assay was therefore evaluated by measuring 0.02 ng (4.1 × 10^6^ DNA copies) and 20 fg (4.1 × 10^3^ copies) of pUC57-bc48 plasmid DNA four times (Fig. 6a). Since quantification was based on the onset time of amplification, we investigated this across the four experiments. The results showed that the deviations of onset time were 8.53% for 0.02 ng and 6.03% for 20 fg DNA (Fig. 6b), an acceptable level of repeatability.

## Conclusion

In view of the harm caused by EP and the inadequacy of existing rapid diagnosis methods, we developed a new real-time qRPA system for the rapid diagnosis of the typical pathogens of EP: *B. caballi* and *T. equi*. The presented qRPA assay, by amplifying the *bc48* or *18S rRNA* gene of *B. caballi* and the *ema-1* gene or *18S rRNA* gene of *T. equi*, showed strict specificity and comparable sensitivities. The developed qRPA strategy has a simpler operating process with a lower constant reaction temperature (39~42 °C) and was more suitable to assay blood specimens in the field as a rapid diagnosis method compared with traditional qPCR. Furthermore, a duplex analysis strategy based on the *bc48* gene of *B. caballi* and the *18S rRNA* gene of *T. equi* was established, and needed only 20 min to amplify the pathogens in horse blood specimens. The qRPA, including the duplex strategy described here, shows great potential for on-site EP screening.

## Materials and Methods

### Materials

Qubit^®^ dsDNA HS Assay kit and Qubit^®^ 2.0 Fluorometer were from Life technologies (Carlsbad, CA). A TwistAmp^®^ exo enzyme pellet including Mg(Ac)_2_ solution (280 mM in water) were purchased from TwistDx Co. (Cambridge, UK). Agencourt AMPure XP beads were obtained from Beckman Coulter (California, USA). All chemicals were of reagent grade. Milli-Q grade (>18 MΩ) water from a Milli-Q water purification system (Merck KGaA, Darmstadt, Germany) was autoclaved for 20 min at 120 °C was used throughout the experiment.

The horse blood samples were obtained from the Hongshan Military Horse Farm of the Beijing Military Region in the Keshiketeng Banner (World Geopark) (Chifeng City, Inner Mongolia) and Heilongjiang Bayi Agricultural University (Daqing, Heilongjiang). EP depends on the seasonal activity of ticks and the highest incidence is at the end of April. Therefore, 30 horse blood samples were taken from tick-bitten horses during this period. The other parasites, *i.e. trypanosoma evansi* (strain STIB 805), *trypanosome brucei* (brucei TREU927), *toxoplasma gondii* (RH), *plasmodium falciparum* (3D7), *theileria hirci* (Dschunkowsky), *theileria sinensis* (Blood extract), *trichinella spiralis inorganic* (Blood extract) were stored in Shenyang Agricultural University (Shenyang, Liaoning). The information of all these samples were listed in Table 1. Horse blood sample collection and processing were performed according to Procedures for Veterinary Clinical Techniques published by China Agriculture Press, and Veterinary Laboratory Biosafety Guidelines approved by the Ministry of Agriculture of the People’s Republic of China.

### DNA extraction procedure

Total DNA was extracted from 200 μl of whole blood using the DNeasy Blood and Tissue Kit (Qiagen, Hilden, Germany) according to the manufacturer’s protocol. Briefly, 20 μl Qiagen Protease was added into the blood sample, then 200 μl Buffer AL was added, followed by a brief vortexing. This mixture was incubated at 56 °C for 10 min with four times up-and-down mixing. After a brief vortexing, 200 μl ethanol was added to the sample, and mixed thoroughly by vortexing. Then the DNA released from the sample was purified with the DNeasy mini spin column with AW1 and AW2 washes, respectively. At last, 100 μl of elution buffer was used to elute the DNA retained on the membrane of the spin column. The eluted DNA was quantified using a Qubit^®^ 2.0 Fluorometer and a Qubit^®^ dsDNA HS Assay kit.

### Primer design

Two genes from *B. caballi* were chosen as the target of the RPA assay: *bc48* (GenBank No. AB017700.1) and *18S rRNA* (GenBank No. Z15104.1). The two genes from *T. equi* were *ema-1* (GenBank No. KC347577.1) and *18S rRNA* (GenBank No. Z15105.1). The primers and probes are listed in Tables 2 and 3. Primers and probes for *bc48 and ema-1* were listed in Table 2, and for *18S rRNA* in Table 3. The forward and reverse primers had 30–35 bp. The probes were labelled with a FAM/ Black Hole Quencher 1 or ROX/Black Hole Quencher 1. All primers and probes were evaluated for biophysical properties and dimer formation by Oligoanalyzer 3.1 (IDT, Leuven, Belgium) and blasted (https://www.ncbi.nlm.nih.gov/tools/primer-blast) against the NCBI nucleotide database to make sure that there was no homology with sequences from another organism.

**Table 2:** Sequences of primers and probes used in the RPA assays for *Theileria equi* and *Babesia caballi*.

Name	Sequences 5′-3′
B. caballi-bc48-F1	CCATCATGGCTCCCAGCGACTCTGTGGGCGACG
B. caballi-bc48-F2	CCGTGTTTCCATCATGGCTCCCAGCGACTCTGT
B. caballi-bc48-R1	CTCATGTCAC TGTTGATCAT ATAGGCATTG GCAGC
B. caballi-bc48-R2	TGTTGATCATATAGGCATTGGCAGCTGAGTCCAC
B. caballi-bc48-P	AGCGACTCTGTGGGCGACGTGACTAAGACCT(FAM-dT)(THF)(BHQ1-dT)TGGCTGCCAGCGAA-C3 spacer
T. equi-ema-F1	CCATTTCGAGCATCCTCGCCGAGGAGGAGAGAC
T. equi-ema-F2	CCCAAGGCCTCTGGAGCTGTCGTCGACTTCCAG
T. equi-ema-R1	TAGACGATGTGCTCCTCGGACTGCTTGTCGATGG
T. equi-ema-R2	CATGAGCAGTGTAGACGATGTGCTCCTCGGACTGC
T. equi-ema-P	ATCCTCGCCGAGGAGGAGAGACCCAAGGCC(ROX-dT)(THF)(BHQ1-dT)GGAGCTGTCGTCGA-C3 spacer

Note: FAM-dT and ROX-dT: thymidine nucleotide carrying Fluorochrome; THF: tetrahydrofuran spacer; BHQ1-dT: thymidine nucleotide carrying Black Hole quencher.

**Table 3:** Sequences of primers and probes used in the exo RPA assays for the *18S rRNA* gene of *Theileria equi* and *Babesia caballi*.

Name	Sequences 5′-3'
B. caballi-18S-F1	ACAGCGACAAGCTGTAGGGAAGTTTAAGGC
B. caballi-18S-F2	CTTCCCTTTTTTTGTTTGGGTTTGCTTCTTAGAG
B. caballi-18S-F3	GGGTTTGCTTCTTAGAGGGACTTTACAGCGACAAG
B. caballi-18S-R1	CCCACACCTTTCGGAGCAGGAAAAACTTAGTGA
B. caballi-18S-R2	CACCTTTCGG AGCAGGAAAAACTTAGTGAATGCA
B. caballi-18S-R3	AGGCAAAACCGACGAATCGGAAAAGCCACGGTCCG
B. caballi-18S-P	ACAAGCTGTAGGGAAGTTTAAGGCAATAACAGG(FAM-dT)(THF)(BHQ1-dT)GTGATGCCCTTAGA-C3 spacer
T. equi-18S-F1	ATAGGGTGTGAGACTTGGTTTCATTTCCGCTTC
T. equi-18S-F2	GTGAGACTTGGTTTCATTTCCGCTTCTTAGAGGG
T. equi-18S-F3	GGTGTGAGACTTGGTTTCATTTCCGCTTCTTAGA
T. equi-18S-R1	ATTACCCAAGCCTCTCAGCCAAGGATACACTC
T. equi-18S-R2	AGCCTCTCAGCCAAGGATACACTCAGTGAATGC
T. equi-18S-P	AGGGACTTTGCGGTCATAAATCGCAAGGAAG(ROX-dT)(THF)(BHQ1-dT)AAGGCAATAACAGG-C3 spacer

Note: FAM-dT and ROX-dT: thymidine nucleotide carrying Fluorochrome; THF: tetrahydrofuran spacer; BHQ1-dT: thymidine nucleotide carrying Black Hole quencher.

### Construction of recombinant plasmid carrying targeting genes of *B. caballi* or and *T. equi*

Sequence of the *bc48* or *18S rRNA* genes from *B. caballi*, and the *ema-1* or *18S rRNA* genes from *T. equi*, were commercially synthesized and cloned into plasmid pUC57 (Genscript, Jiangsu, China), respectively. The prepared recombinant plasmids termed pUC57-bc48, pUC57-Bc18S, pUC57-ema and pUC57-Te18S, respectively. Then, plasmids were replicated in *E. coli* DH5α cells, extracted and purified using an endotoxin-free Plasmid Maxiprep Kit. After that, plasmid DNA was quantified using a Qubit^®^ 2.0 Fluorometer and a Qubit^®^ dsDNA HS Assay kit.

### Development of RPA assay

The RPA assay was performed based on the manual instructions. In brief, each assay was performed in a test tube filled with TwistAmp^TM^ exonuclease and 50 μl reaction mixture. In process, the enzyme pellet was rehydrated by 46.5 μL of a master-mix containing rehydration buffer (29.5 μL), nuclease-free water (12.2 μL), 10 μM forward and reverse primer (each 2.1 μL) and 10 μM probe (0.6 μL). Next, 2 μL of plasmid DNA or DNA specimen extracted from parasites was added to each reaction, with a final concentration of 420 nM for each primer and 120 nM for probe. Last, 2.5 μL of Mg(Ac)_2_ (280 mM) was added to the lid of each tube. All reactions were simultaneously initiated by centrifuging the magnesium acetate into the reaction mixture and transferring the tubes to a 39 °C heat block for 30 min. Fluorometric data were collected every 10 s. Referring to the previous works, the onset time of amplification was used to calculate the linearity and limit of detection, which was more practical than fluorescence intensity and helpful to improve the repeatability of the real-time RPA assay^55,56^.

### Evaluation of specificity and sensitivity

Sensitivity of the RPA assay were tested using pure recombinant plasmid genomic DNA, which allowed accurate determination of the detection limit in terms of copy numbers. A ten-fold serial dilution of recombinant plasmid DNA, ranging from 2.0 ng to 0.02 fg, was used as standard to determine sensitivity level, establish amplification efficiency and resolve the limit of detection. The DNA copy number of 2.0 ng of the pUC57-bc48 plasmid is equal to 4.1 × 10^8^ copies; 2.0 ng of pUC57-ema equates to 5.26 × 10^8^ copies; 2.0 ng of pUC57-Bc18S equates to 4.21 × 10^8^ copies; and 2.0 ng of pUC57-Te18S is 4.16 × 10^8^ copies. For specificity, the interfering DNA samples from *trypanosoma evansi* (strain STIB 805), *trypanosome brucei* (brucei TREU927), *toxoplasma gondii* (RH), *plasmodium falciparum* (3D7), *theileria hirci* (Dschunkowsky), *theileria sinensis* (Blood extract), *trichinella spiralis inorganic* (Blood extract), were evaluated using the developed duplex RPA assay. The onset time of amplification was plotted against the concentration of total DNA.

As a comparison, real-time PCR assay was also performed. Primers and probes reported in a previous duplex qPCR assay of EP^26,28^ (Table 4) were synthesized by Sangon Biotech (Shanghai, China). The PCR reactions were run on a Light Cycler 480 (Roche, USA) using a TaqMan Gene Expression Master Mix (Thermo Fisher Scientific, USA). The cycling protocol consisted of the initial activation cycle at 95 °C for 10 min, followed by 45 cycles of 30 s at 95 °C, 30 s at 57 °C and 20 s at 72 °C with fluorescence data acquisition after the elongation step. Negative controls consisted of nuclease-free water and DNA extracted from the blood of an EP-free horse.

**Table 4:** Nucleotide sequences of primers and probes used in the duplex qPCR assay.

Name	Sequences 5′-3′
Bc_18SF402	GTAATTGGAATGATGGCGACTTAA
Bc_18SR4596	CGCTATTGGAGCTGGAATTACC
Bc_18SP	VIC-CCTCGCCAGAGTAA-MGB-NFQ
Te_EMA1-F	CTGACTACAAGGTYGTATAC
Te_EMA1-R	TGTCGTCACTT AGTAAAATAGA
Te_EMA1-P	6-FAM-TTCTCCGTCTATGGCGCA-MGB-NFQ

Abbreviations: MGB, minor groove binder; NFQ, non-fluorescent quencher

### Ethical statement

The Ethical Committee of Shenyang Agricultural University approved the laboratory animal experiments (permit no. SYXK <Liao> 2011–0001).

## Supplementary information

Additional file.
