# Supplementary material for: Rapid isothermal duplex real-time recombinase polymerase amplification (RPA) assay for the diagnosis of equine piroplasmosis
Source: Sci Rep. 2020 Mar 5;10:4096. doi: 10.1038/s41598-020-60997-1 (PMC7058082; doi:10.1038/s41598-020-60997-1)
Supplement: Supplementary file 1 — Additional file. [file 41598_2020_60997_MOESM1_ESM.docx]

**Rapid isothermal duplex real-time recombinase polymerase amplification (RPA) assay for the diagnosis of equine piroplasmosis**

**Rong Lei^2#^, Xinyi Wang^1,3#^, Di Zhang^3^, Yize Liu^1^, Qijun Chen^1^, Ning Jiang^1*^**

^1^ Key Laboratory of Livestock Infectious Diseases in Northeast China, Ministry of Education, Key Laboratory of Zoonosis, Shenyang Agricultural University, Shenyang 110866, China

^2^ Chinese Academy of Inspection and Quarantine, Beijing, 100176, China

^3^ College of Land and Environment, Shenyang Agricultural University, Shenyang 110866, China

^*^ corresponding author: jiangning@syau.edu.cn

^#^ these authors contributed equally to this work

**Additional files
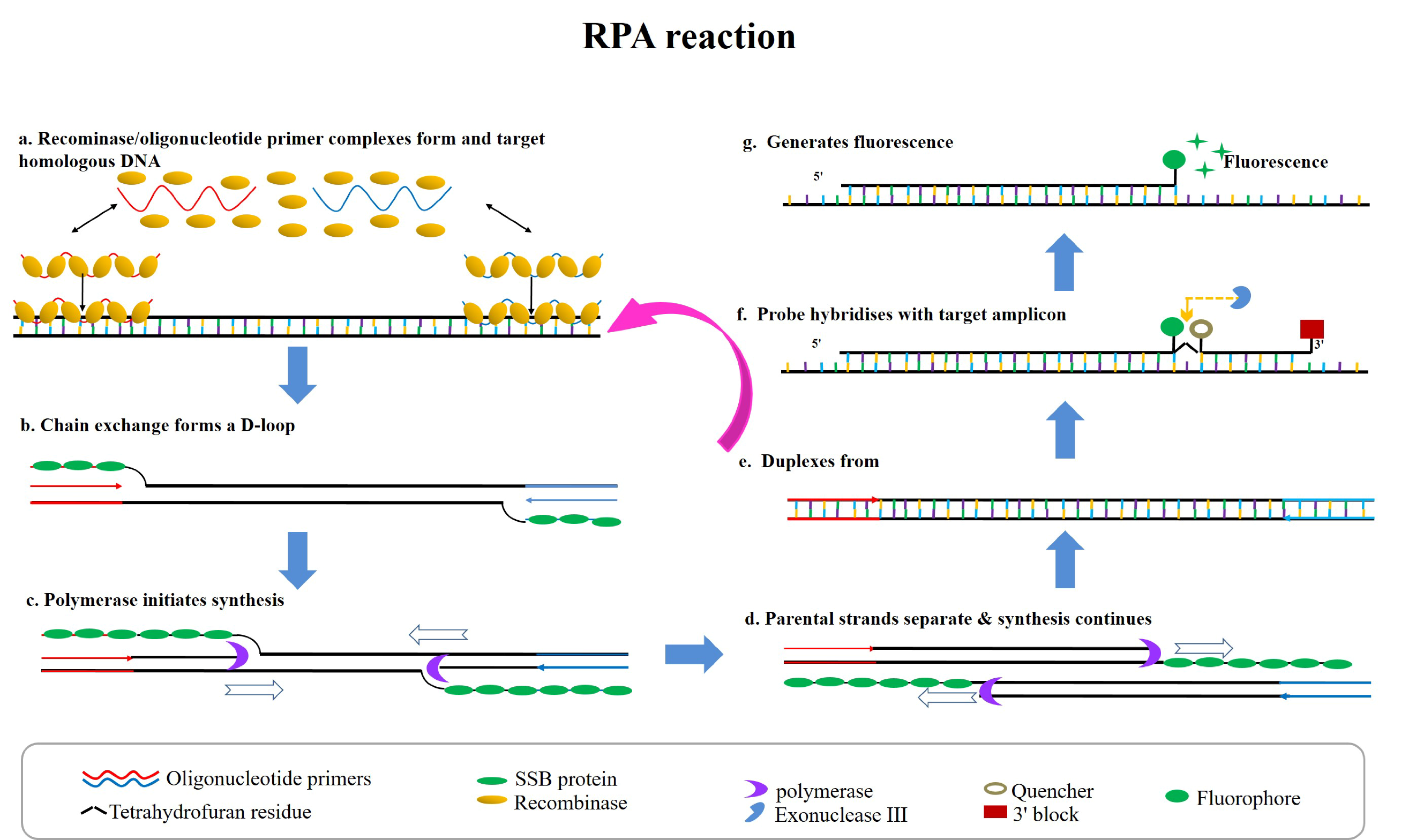
**

**Figure S1** RPA reaction principle. (a) Recombinase/primer form, a protein-DNA complex, can target homologous DNA sequences. (b)-(e) Once the recombinase/primer complex attaches homologous sequences, strand exchange reactions occur, and synthesis of DNA double strands is initiated, and cycle execution of recombinase/oligonucleotide primer complexes contributes to the exponential amplification of the target region strands on the template. (f)-(g) The *exo* probe technology, exonuclease III and reverse transcriptase combine to read fluorescence data in real time.


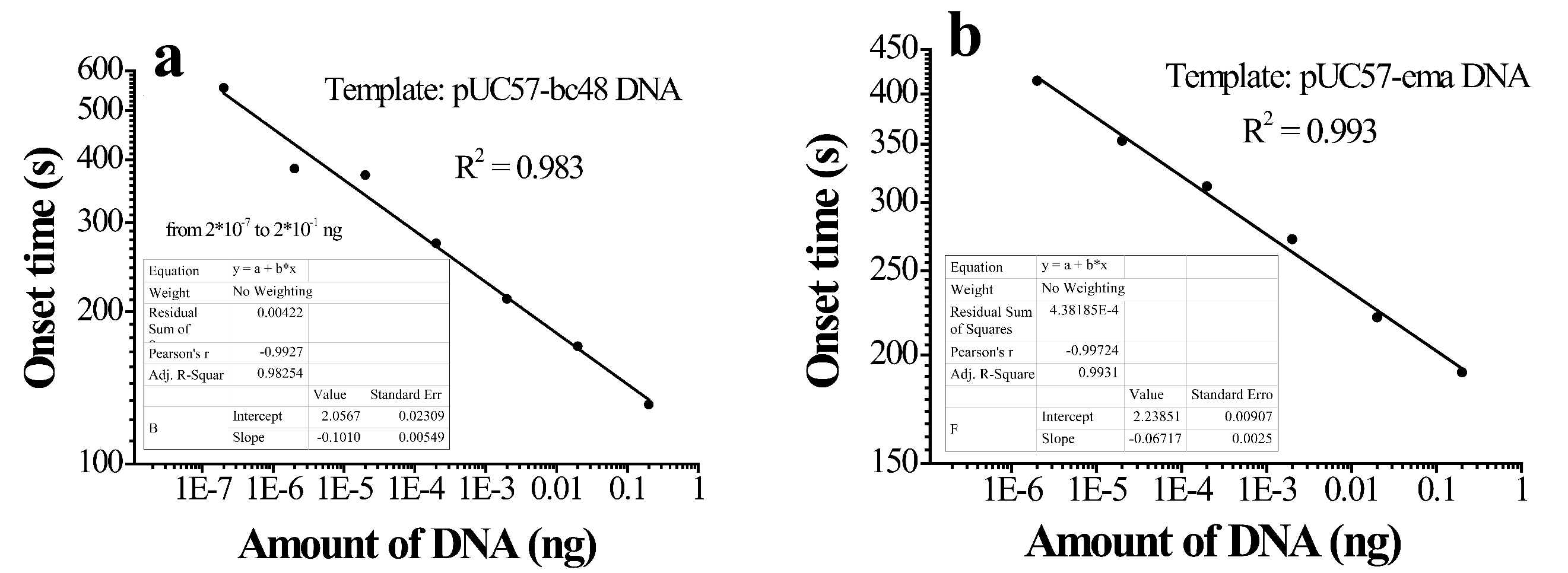


**Figure S2** Standard curve plots of the log of initial DNA amount and the onset time of amplification using RPA assay for pUC57-bc48 plasmid DNA (a) and pUC57-ema plasmid DNA (b).


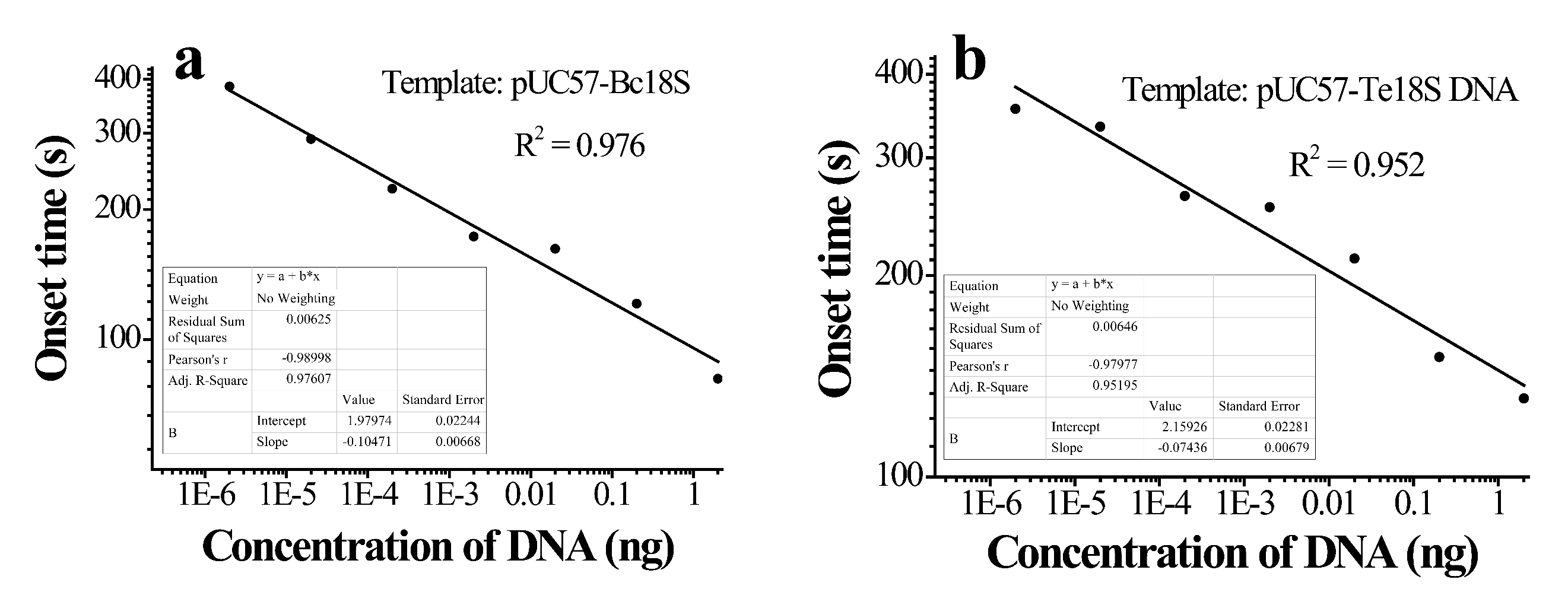


**Figure S3** Standard curve plots of the log of initial DNA amount and the onset time of amplification using RPA assay for pUC57-Bc18S plasmid DNA (a) and pUC57-Te18S plasmid DNA (b).


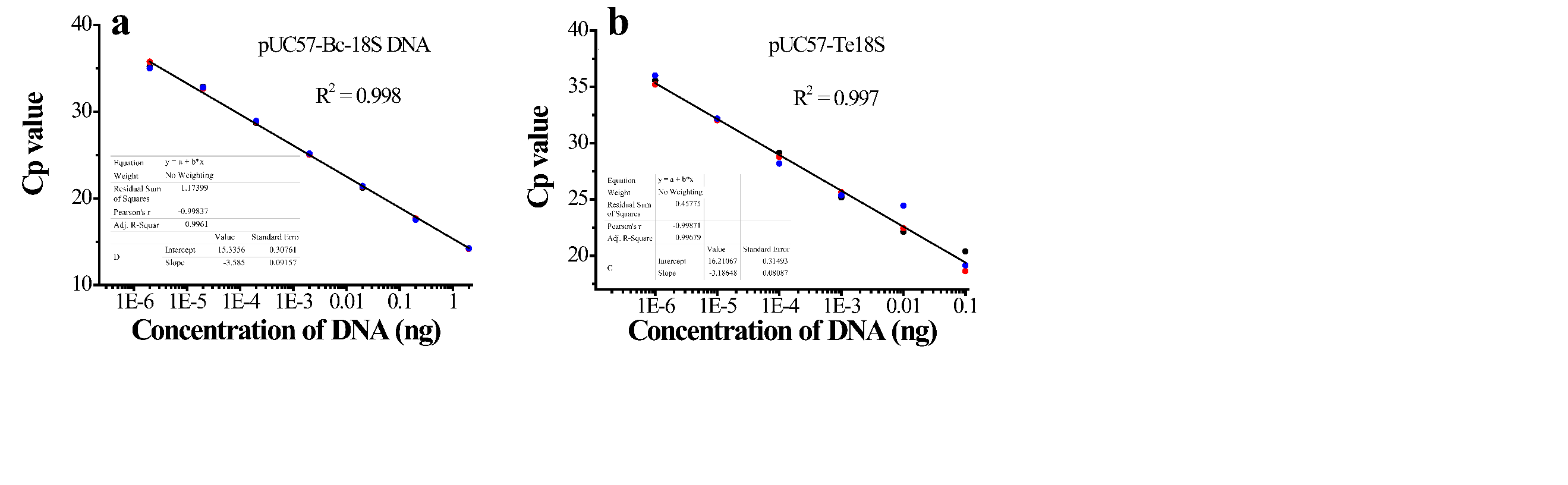


**Figure S4** Standard curve plots of the log of initial DNA amount and the onset time of amplification using real-time PCR assay for pUC57-Bc18S plasmid DNA (a) and pUC57-ema plasmid DNA (b).
